# Genome Sequence of *Christensenella minuta* DSM 22607^T^

**DOI:** 10.1128/genomeA.01451-16

**Published:** 2017-01-12

**Authors:** Bruce A. Rosa, Kymberlie Hallsworth-Pepin, John Martin, Aye Wollam, Makedonka Mitreva

**Affiliations:** a The McDonnell Genome Institute, Washington University School of Medicine, St. Louis, Missouri, USA; b Department of Medicine, Washington University School of Medicine, St. Louis, Missouri, USA

## Abstract

Obesity influences and is influenced by the human gut microbiome. Here, we present the genome of *Christensenella minuta*, a highly heritable bacterial species which has been found to be strongly associated with obesity through an unknown biological mechanism. This novel genome provides a valuable resource for future obesity therapeutic studies.

## GENOME ANNOUNCEMENT

The gut microbiome is acquired naturally from birth, after which it is affected by host genetics, environmental factors, and diet (1). Due to its widespread and significant effects on human health, modulation of the gut microbiome is an emerging therapeutic paradigm, particularly for obesity, diabetes, and inflammatory bowel diseases (2).

Through complex interactions with host metabolism, the gut microbiome both influences and is influenced by the obesity phenotype (3–5). For example,* Firmicutes* bacteria are more abundant at the expense of *Bacteroides* bacteria, both as a result of a high fat/high sugar diet (6) and as a consequence of host genetic obesity due to leptin deficiency (7). However, microbiome-targeted therapeutic efforts for obesity have been hampered by a lack of understanding of the interactions between host genetics and the microbiome, as well as the complexity and diversity of the microbiome (2). Recently, *Christensenella minuta* (the first described member of the *Christensenellaceae* family [8]) was described as being extremely highly heritable and as promoting a lean host phenotype through an unknown biological mechanism, a finding which was experimentally verified using transplantation techniques in germ-free mice (3).

In order to facilitate further research into identifying the biological mechanisms underlying the important role this organism plays in obesity prevention, here we describe the first genome sequence of *Christensenella minuta* DSM 22607^T^.

Genomic DNA was obtained from the DSMZ repository and sequenced using the Illumina HiSeq 2000, to a depth of 115×. *De novo* assembly of the genome was conducted using the One Button Velvet assembly pipeline version 1.1.06 (9). Gene annotation was performed using both *ab initio* and evidence-based (BLAST) predictions. Coding sequences were predicted using GeneMark and Glimmer3 (10, 11). Intergenic regions not identified by GeneMark and Glimmer3 were searched by BLAST in NCBI’s nonredundant bacterial (NR) database. The best prediction for each open reading frame was selected by evaluating all predictions against the best evidence (NR and Pfam [12]) and resolving overlaps between adjacent coding genes. tRNA genes were determined using tRNAscan-SE (13) and noncoding RNA genes by RNAmmer (14) and Rfam (15). We performed a screen for core genes, as defined by the HMP project (16), on many of the assemblies to test for completeness of the genome. Metabolic pathways and subcellular localization were predicted using KEGG (17) and psortB (18), respectively, and functional domains were evaluated using Interproscan (19) (used to infer gene ontology [GO] terms [20, 21]) and Pfam (22). A total of 2,487 genes (79.7% of all genes) had some predicted functional annotation, with 66.1% being assigned to at least one of 949 unique GO term annotations, 74.4% containing at least one of 2,822 unique InterPro domains, and 69.5% containing at least one of 1,412 unique Pfam domains.

### Accession number(s).

This whole-genome shotgun project has been deposited in DDBJ/ENA/GenBank under the accession number LSZW00000000. The version described in this paper is the first version, LSZW01000000.
